# Preferences for healthcare decisional control in older people with chronic kidney disease in the UK indicate strong inclinations towards active and collaborative approaches

**DOI:** 10.1093/ckj/sfag010

**Published:** 2026-01-21

**Authors:** Sanjana Mathew, Fergus J Caskey, Leila Rooshenas, Rachael L Morton, Lucy E Selman, Joanna Coast, Barnaby Hole

**Affiliations:** Department of Renal Medicine, Southmead Hospital, North Bristol NHS Trust, Bristol, UK; Department of Renal Medicine, Southmead Hospital, North Bristol NHS Trust, Bristol, UK; Bristol Medical School, University of Bristol, Bristol, UK; Bristol Medical School, University of Bristol, Bristol, UK; NHMRC Clinical Trials Centre, University of Sydney, Sydney, NSW, Australia; Bristol Medical School, University of Bristol, Bristol, UK; Bristol Medical School, University of Bristol, Bristol, UK; Department of Renal Medicine, Southmead Hospital, North Bristol NHS Trust, Bristol, UK; Bristol Medical School, University of Bristol, Bristol, UK

To the Editor,

Individuals with chronic kidney disease (CKD) are encouraged to prepare for possible kidney failure. UK guidelines advocate shared decision-making (SDM) as a cornerstone of patient-centred care, defining it as a collaborative process in which patients and clinicians work together to reach healthcare decisions [1]. While Western research overall supports SDM through evidence that most patients prefer decisional autonomy [2], evidence is lacking in the underresearched cohort of older adults with CKD and regarding factors influencing these preferences. We present findings from a UK-wide survey exploring decision-making preferences among this cohort and associations with socio-economic status and well-being.

This work formed part of a larger study reported elsewhere [3]. Between November 2021 and June 2024, individuals >65 years of age with an estimated glomerular filtration rate (eGFR) ≤20 ml/min/1.73 m^2^ were recruited from 23 kidney centres across the UK. Participants completed a postal questionnaire and were required to understand English and have no prior experience of outpatient dialysis or kidney transplantation. Decision-making preferences were assessed using the Control Preferences Scale (CPS), a validated measure in which respondents select one of five roles, later grouped into three categories: ‘active’ (patient led), ‘collaborative’ (SDM) and ‘passive’ (clinician led). Capability and well-being were measured using the ICEpop CAPability measure for Older people (ICECAP-O), with lower scores indicating lower well-being. Socio-economic status was derived from postcode-linked Index of Multiple Deprivation (IMD) deciles, with lower scores indicating greater deprivation.

Ordinal logistic regression in Stata, version 18.5 (StataCorp, College Station, TX, USA) was used to explore associations between decision-making preference and demographic factors such as well-being, deprivation, age, gender and ethnicity.

Of 524 eligible participants, 368 completed the CPS (70.2%). Participants’ mean age was 77 years; 66% were male and 82% were White. Overall, 57% preferred an active role in decision-making, 33% preferred collaborative and only 10% preferred a passive role. In univariable analysis, higher well-being (ICECAP-O *r* = −3.61, *P* < .01) and lower deprivation (IMD *r* = −0.10, *P* < .01) were significantly associated with more active decision-making preferences. In multivariable analysis, higher well-being (ICECAPO *r* = −3.48, *P* < .01) and lower deprivation (IMD *r* = −0.12, *P* = .01) remained independently associated with active preferences (Fig. 1).

**Figure 1:**
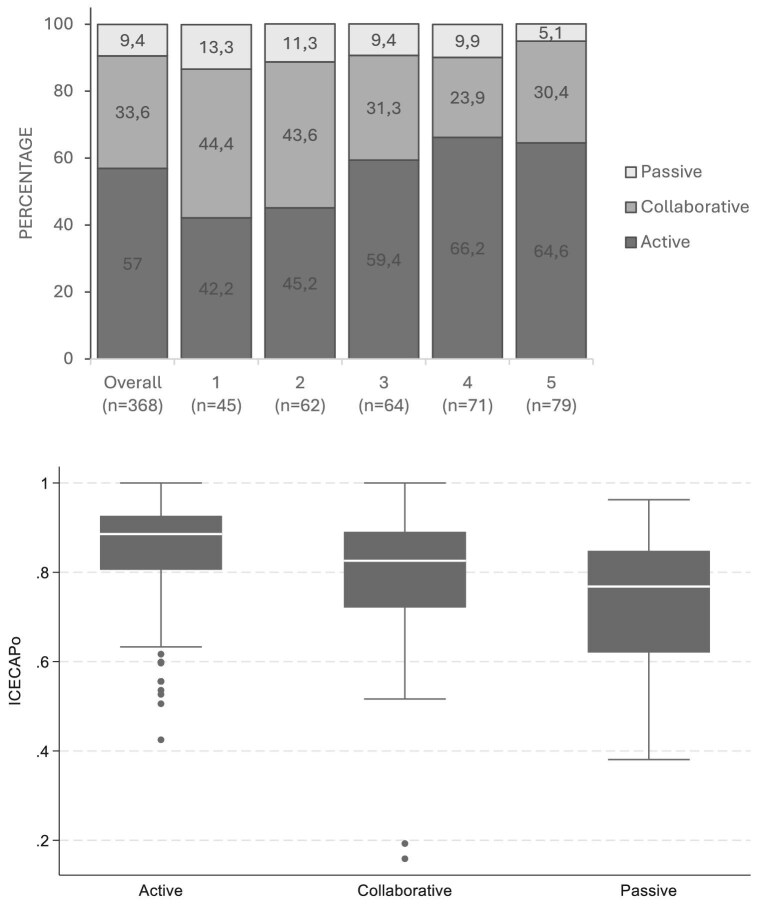
Distribution of CPS categories across IMD quintiles (top panel) and distribution of ICECAPO scores in each CPS category (bottom panel).

These findings align with similar distributions of preferences reported in US populations, where the majority of older adults with CKD favour active or shared decision-making [2]. However, we are not aware of studies linking higher socio-economic status or well-being with active decision-making preferences. This link between well-being and decisional control is not necessarily causal. Individuals experiencing lower well-being or socio-economic disadvantage may defer more to clinicians; alternatively, those who relinquish control may subsequently experience poorer outcomes that further reduce well-being. Supporting this, evidence indicates that dialysis patients with treatment regret often recall decisions as being clinician-driven [4].

Strengths of this study include recruitment of a clinically relevant population facing imminent, preference-sensitive decisions and inclusion of participants from a wide range of socio-economic backgrounds. Limitations include a response rate of 70%, raising the possibility that individuals with particular preferences were underrepresented. White and less-deprived participants were overrepresented compared with the broader UK kidney failure population [5]. Additionally, CPS responses are hypothetical and may not reflect real-world behaviour.

In conclusion, few older people with advanced CKD in the UK prefer to relinquish decisional control. Associations between socio-economic status, well-being and decision-making preferences highlight an underexplored dimension of nephrology and raise an important question: should clinicians encourage active decision-making or prioritise supporting individuals to exercise the level of control that matches their preferences? Evidence suggests we may currently fall short of both aims [4]. Clinicians should seek to identify and respect patients’ decision-making preferences, while remaining mindful that deference to clinicians may risk reinforcing existing health and socio-economic inequalities.

## Data Availability

Data are available on request to the corresponding author.
